# Supplementary material for: Stronger net selection on males across animals
Source: eLife. 2021 Nov 17;10:e68316. doi: 10.7554/eLife.68316 (PMC8598160; doi:10.7554/eLife.68316)
Supplement: Supplementary file 1. [file elife-68316-supp1.docx]

**Supplementary File 1. Results of PGLMMs testing for sex by mating system interaction on phenotypic (*CV_P_*) and genetic (*CV_G_*) coefficient of variation.** Results are shown for reproductive success (RS) and lifespan (LS). Estimates are shown as posterior means with 95% Highest Posterior Density (HPD) intervals. *P*_MCMC_ is the probability of the posteriors including zero.

| Response | Variance  component | Predictor | Estimate | | | *P*_MCMC_ |
| --- | --- | --- | --- | --- | --- | --- |
| RS | *CV*_P_ | Sex | -0.012 | (-0.168, | 0.150) | 0.878 |
|  |  | Mating system | 0.025 | (-0.344, | 0.403) | 0.916 |
|  |  | Sex by Mating system | 0.324 | (0.142, | 0.506) | < 0.001 |
|  | *CV*_G_ | Sex | -0.015 | (-0.097, | 0.069) | 0.712 |
|  |  | Mating system | -0.011 | (-0.166, | 0.151) | 0.894 |
|  |  | Sex by Mating system | 0.134 | (0.039, | 0.229) | 0.006 |
| LS | *CV*_P_ | Sex | 0.027 | (-0.029, | 0.080) | 0.325 |
|  |  | Mating system | 0.100 | (-0.224, | 0.441) | 0.575 |
|  |  | Sex by Mating system | -0.041 | (-0.103, | 0.020) | 0.192 |
|  | *CV*_G_ | Sex | 0.050 | (0.003, | 0.097) | 0.039 |
|  |  | Mating system | -0.035 | (-0.167, | 0.096) | 0.583 |
|  |  | Sex by Mating system | -0.043 | (-0.097, | 0.010) | 0.116 |
