# Supplementary material for: Stronger net selection on males across animals
Source: eLife. 2021 Nov 17;10:e68316. doi: 10.7554/eLife.68316 (PMC8598160; doi:10.7554/eLife.68316)
Supplement: Supplementary file 2. [file elife-68316-supp2.docx]

**Supplementary File 2.** **Phylogenetic General Linear Mixed Effect Models testing for an effect of sex on phenotypic (*CV*_P_) and genetic (*CV*_G_) coefficient of variation for a reduced dataset including only vertebrates.** Results are shown for reproductive success (RS) and lifespan (LS) for models across mating systems and when ran separately for socially monogamous and polygamous species. Estimates are shown as posterior means with 95% Highest Posterior Density (HPD) intervals, with positive values indicating a male bias. *P*_MCMC_ is the probability of the posteriors including zero.The variance explained by sex is given as the marginal *R*^2^ and the phylogenetic signal is reported as *H*^2^.

| Response | Variance  component | Sex effect estimate | | | *P*_MCMC_ | Marginal *R*^2^ | | | Phylogenetic *H*^2^ | | |
| --- | --- | --- | --- | --- | --- | --- | --- | --- | --- | --- | --- |
| Across mating systems | | |  |  |  |  |  |  |  |  |  |
| RS | CV_P_ | 0.187 | (0.013, | 0.361) | 0.037 | 0.03 | (0.00, | 0.09) | 0.13 | (0.00, | 0.43) |
|  | CV_G_ | 0.084 | (0.001, | 0.165) | 0.044 | 0.04 | (0.00, | 0.10) | 0.24 | (0.02, | 0.61) |
| LS | CV_P_ | -0.003 | (-0.050, | 0.044) | 0.884 | 0.00 | (0.00, | 0.01) | 0.37 | (0.01, | 0.90) |
|  | CV_G_ | 0.025 | (-0.038, | 0.087) | 0.417 | 0.01 | (0.00, | 0.04) | 0.44 | (0.06, | 0.84) |
| Monogamy |  |  |  |  |  |  |  |  |  |  |  |
| RS | CV_P_ | -0.012 | (-0.065, | 0.035) | 0.594 | 0.00 | (0.00, | 0.00) | 0.37 | (0.01, | 0.96) |
|  | CV_G_ | -0.014 | (-0.079, | 0.047) | 0.632 | 0.01 | (0.00, | 0.02) | 0.43 | (0.05, | 0.91) |
| LS | CV_P_ | 0.027 | (-0.031, | 0.077) | 0.281 | 0.00 | (0.00, | 0.02) | 0.37 | (0.01, | 0.87) |
|  | CV_G_ | 0.050 | (-0.029, | 0.127) | 0.179 | 0.02 | (0.00, | 0.07) | 0.46 | (0.07, | 0.90) |
| Polygamy |  |  |  |  |  |  |  |  |  |  |  |
| RS | CV_P_ | 0.564 | (0.151, | 0.993) | 0.016 | 0.15 | (0.00, | 0.34) | 0.16 | (0.00, | 0.60) |
|  | CV_G_ | 0.270 | (0.116, | 0.433) | 0.003 | 0.16 | (0.00, | 0.34) | 0.23 | (0.00, | 0.69) |
| LS | CV_P_ | -0.071 | (-0.218, | 0.083) | 0.233 | 0.01 | (0.00, | 0.04) | 0.53 | (0.02, | 1.00) |
|  | CV_G_ | -0.031 | (-0.214, | 0.165) | 0.658 | 0.02 | (0.00, | 0.09) | 0.41 | (0.00, | 0.94) |
