# Supplementary material for: Stronger net selection on males across animals
Source: eLife. 2021 Nov 17;10:e68316. doi: 10.7554/eLife.68316 (PMC8598160; doi:10.7554/eLife.68316)
Supplement: Supplementary file 3. [file elife-68316-supp3.docx]

**Supplementary File 3. Results of PGLMMs testing for sex by mating system interaction on phenotypic (*CV_P_*) and genetic (*CV_G_*) coefficient of variation obtained from a reduced dataset including only vertebrates.** Results are shown for reproductive success (RS) and lifespan (LS). Estimates are shown as posterior means with 95% Highest Posterior Density (HPD) intervals. *P*_MCMC_ is the probability of the posteriors including zero.

| Response | Variance  component | Predictor | Estimate | | | *P*_MCMC_ |
| --- | --- | --- | --- | --- | --- | --- |
| RS | CV_P_ | Sex | -0.012 | (-0.166, | 0.151) | 0.871 |
|  |  | Mating system | 0.065 | (-0.419, | 0.573) | 0.798 |
|  |  | Sex by Mating system | 0.576 | (0.315, | 0.848) | < 0.001 |
|  | CV_G_ | Sex | -0.015 | (-0.088, | 0.063) | 0.692 |
|  |  | Mating system | -0.111 | (-0.312, | 0.102) | 0.271 |
|  |  | Sex by Mating system | 0.285 | (0.158, | 0.412) | < 0.001 |
| LS | CV_P_ | Sex | 0.027 | (-0.024, | 0.073) | 0.244 |
|  |  | Mating system | 0.252 | (-0.278, | 0.779) | 0.305 |
|  |  | Sex by Mating system | -0.097 | (-0.184, | -0.012) | 0.032 |
|  | CV_G_ | Sex | 0.049 | (-0.026, | 0.125) | 0.179 |
|  |  | Mating system | -0.017 | (-0.269, | 0.245) | 0.875 |
|  |  | Sex by Mating system | -0.081 | (-0.217, | 0.056) | 0.221 |
