# Supplementary material for: Stronger net selection on males across animals
Source: eLife. 2021 Nov 17;10:e68316. doi: 10.7554/eLife.68316 (PMC8598160; doi:10.7554/eLife.68316)
Supplement: Supplementary file 4. [file elife-68316-supp4.docx]

**Supplementary File 4. Results of PGLMMs testing for the effect of sex, study type (lab *versus* field studies) and their interaction on phenotypic (*CV_P_*) and genetic (*CV_G_*) coefficients of variation.** Results are shown for reproductive success (RS) and lifespan (LS). Estimates are shown as posterior means with 95% Highest Posterior Density (HPD) intervals. *P*_MCMC_ is the probability of the posteriors including zero.

| Response | Variance  component | Predictor | Estimate | | | P_MCMC_ |
| --- | --- | --- | --- | --- | --- | --- |
| RS | CV_P_ | Sex | 0.187 | (0.047, | 0.333) | 0.011 |
|  |  | Study type | -0.301 | (-0.720, | 0.134) | 0.138 |
|  |  | Sex by Study type | 0.073 | (-0.098, | 0.262) | 0.425 |
|  | CV_G_ | Sex | 0.085 | (0.013, | 0.155) | 0.020 |
|  |  | Study type | 0.134 | (-0.182, | 0.448) | 0.344 |
|  |  | Sex by Study type | 0.003 | (-0.088, | 0.091) | 0.930 |
| LS | CV_P_ | Sex | -0.003 | (-0.050, | 0.042) | 0.894 |
|  |  | Study type | -0.220 | (-0.694, | 0.294) | 0.265 |
|  |  | Sex by Study type | -0.002 | (-0.059, | 0.053) | 0.936 |
|  | CV_G_ | Sex | 0.024 | (-0.016, | 0.064) | 0.232 |
|  |  | Study type | 0.024 | (-0.294, | 0.343) | 0.870 |
|  |  | Sex by Study type | -0.012 | (-0.060, | 0.038) | 0.646 |
