# Supplementary material for: Stronger net selection on males across animals
Source: eLife. 2021 Nov 17;10:e68316. doi: 10.7554/eLife.68316 (PMC8598160; doi:10.7554/eLife.68316)
Supplement: Supplementary file 5. [file elife-68316-supp5.docx]

**Supplementary File 5. Results of PGLMMs testing for the effect of sex, V_G_ estimate (additive *versus* total) and their interaction on genetic (*CV_G_*) coefficient of variation.** Results are shown for reproductive success (RS) and lifespan (LS). Estimates are shown as posterior means with 95% Highest Posterior Density (HPD) intervals. *P*_MCMC_ is the probability of the posteriors including zero.

| Response | Variance  component | Predictor | Estimate | | | P_MCMC_ |
| --- | --- | --- | --- | --- | --- | --- |
| RS | CV_G_ | Sex | 0.118 | (0.060, | 0.180) | < 0.001 |
|  |  | V_G_ estimate | -0.030 | (-0.170, | 0.120) | 0.695 |
|  |  | Sex by V_G_ estimate | -0.064 | (-0.147, | 0.021) | 0.139 |
| LS | CV_G_ | Sex | 0.007 | (-0.029, | 0.046) | 0.693 |
|  |  | V_G_ estimate | 0.006 | (-0.111, | 0.126) | 0.920 |
|  |  | Sex by V_G_ estimate | 0.015 | (-0.032, | 0.063) | 0.516 |
