# Supplementary material for: Stronger net selection on males across animals
Source: eLife. 2021 Nov 17;10:e68316. doi: 10.7554/eLife.68316 (PMC8598160; doi:10.7554/eLife.68316)
Supplement: Supplementary file 6. [file elife-68316-supp6.docx]

**Supplementary File 6. Results of PGLMMs testing for the effect of sex, RS estimate (temporal *versus* lifetime reproductive success) and their interaction on phenotypic (*CV_P_*) and genetic (*CV_G_*) coefficients of variation.** Estimates are shown as posterior means with 95% Highest Posterior Density (HPD) intervals. *P*_MCMC_ is the probability of the posteriors including zero.

| Response | Variance  component | Predictor | Estimate | | | P_MCMC_ |
| --- | --- | --- | --- | --- | --- | --- |
| RS | CV_P_ | Sex | 0.312 | 0.192 | 0.436 | < 0.001 |
|  |  | RS estimate | -0.192 | -0.447 | 0.051 | 0.124 |
|  |  | Sex by RS estimate | -0.152 | -0.314 | 0.027 | 0.083 |
|  | CV_G_ | Sex | 0.122 | 0.062 | 0.182 | < 0.001 |
|  |  | RS estimate | 0.041 | -0.085 | 0.159 | 0.502 |
|  |  | Sex by RS estimate | -0.069 | -0.152 | 0.016 | 0.107 |
