# Supplementary material for: Stronger net selection on males across animals
Source: eLife. 2021 Nov 17;10:e68316. doi: 10.7554/eLife.68316 (PMC8598160; doi:10.7554/eLife.68316)
Supplement: Supplementary file 7. [file elife-68316-supp7.docx]

### **Supplementary File 7.** Analysis of cross-sex genetic correlations.

Theory predicting a positive effect of sexual selection on adaptation is based on the additional assumption that alleles favored in males must also be beneficial in females, which, if true, may manifest in a positive genetic correlation between male and female fitness. Estimates of such cross-sex genetic correlations are scarce and a previous comparative study included only few correlation of fitness components relative to morphological, physiological and behavioral traits (Poissant *et al.* 2010).

In an explorative meta-analysis, we tested for an overall positive genetic cross-sex correlation in reproductive success and lifespan based on estimates provided in the primary studies obtained from our systematic literature search. Only few primary studies reported cross-sex correlations, which led to small samples sizes for reproductive success (*N* = 25) and lifespan (*N* = 19), and is also reflected in low numbers of sampled species (reproductive success: *N*_Species_ = 10; lifespan: *N*_Species_ = 5).

We used phylogenetically informed meta-analyses by running PGLMMs with the *MCMCglmm* R package (using same specifications as for models reported in the main text), in which Pearson correlation coefficients (*r*) were defined as response variable weighted by the inverse of its variance. Models included study identifier and phylogeny as random effect terms. We found support for positive cross-sex genetic correlation for lifespan (PGLMM: global *r* = 0.548, 95% CI = 0.185 – 0.918, *P* = 0.020) but not for reproductive success (PGLMM: global *r* = 0.024, 95% CI = -0.242 – 0.244, *P* = 0.816). In conclusion, our limited dataset does not support a positive cross-sex genetic correlation for reproductive success.
