# Supplementary material for: Stronger net selection on males across animals
Source: eLife. 2021 Nov 17;10:e68316. doi: 10.7554/eLife.68316 (PMC8598160; doi:10.7554/eLife.68316)
Supplement: Supplementary file 8. [file elife-68316-supp8.docx]

### **Supplementary File 8.** Search terms and list of primary studies.

Systematic literature search was carried out in the Web of Science Core Collection (Clarivate) using the following search terms:

TS=((sex* OR (male AND female) OR (man AND woman) OR “sex diff*” OR “gender diff*” OR sex-specific OR intersex* OR inter-sex* OR cross-sex* OR “across sex*” OR “between sex*” OR “between-sex*” OR “sex-limited”) AND (fitness OR "reproductive success" OR survival OR longevity OR lifespan OR “life span”) AND ("intra-locus sexual conflict" OR “intralocus sexual conflict” OR “sexually antagonistic genetic” OR "genetic co*” OR heritability OR "genetic varia*" OR "quantitative genetics" OR “genetic architecture” OR evolvability))

The list below encompasses all 52 published primary studies included in the comparative analyses. It does not comprise three unpublished studies that have also been included (Abbott, J., and A. Norden. in prep.; Janicke, T., E. Chapuis, S. Meconcelli, N. Bonel, and P. David. in prep.; Moiron M, Charmantier A, Bouwhuis S. in prep.).

Archer, C. R., F. Zajitschek, S. K. Sakaluk, N. J. Royle, and J. Hunt. 2012. Sexual selection affects the evolution of lifespan and ageing in the decorated cricket *Gryllodes sigillatus*. Evolution 66:3088-3100.

Berger, D., K. Grieshop, M. I. Lind, J. Goenaga, A. A. Maklakov, and G. Arnqvist. 2014. Intralocus sexual conflict and environmental stress. Evolution 68:2184-2196.

Bolund, E., S. Bouwhuis, J. E. Pettay, and V. Lummaa. 2013. Divergent selection on, but no genetic conflict over, female and male timing and rate of reproduction in a human population. Proceedings of the Royal Society B-Biological Sciences 280.

Brommer, J. E., M. Kirkpatrick, A. Qvarnstrom, and L. Gustafsson. 2007. The intersexual genetic correlation for lifetime fitness in the wild and its implications for sexual selection. Plos One 2.

Calsbeek, R., M. C. Duryea, D. Goedert, P. Bergeron, and R. M. Cox. 2015. Intralocus sexual conflict, adaptive sex allocation, and the heritability of fitness. Journal of Evolutionary Biology 28:1975-1985.

Collet, J. M., S. Fuentes, J. Hesketh, M. S. Hill, P. Innocenti, E. H. Morrow, K. Fowler et al. 2016. Rapid evolution of the intersexual genetic correlation for fitness in *Drosophila melanogaster*. Evolution 70:781-795.

Coltman, D. W., P. O'Donoghue, J. T. Hogg, and M. Festa-Bianchet. 2005. Selection and genetic (co)variance in bighorn sheep. Evolution 59:1372-1382.

Delcourt, M., M. W. Blows, and H. D. Rundle. 2009. Sexually antagonistic genetic variance for fitness in an ancestral and a novel environment. Proceedings of the Royal Society B-Biological Sciences 276:2009-2014.

Duffy, E., C. R. Archer, M. D. Sharma, M. Prus, R. A. Joag, J. Radwan, N. Wedell et al. 2019. Wolbachia infection can bias estimates of intralocus sexual conflict. Ecology and Evolution 9:328-338.

Duffy, E., R. Joag, J. Radwan, N. Wedell, and D. J. Hosken. 2014. Inbreeding alters intersexual fitness correlations in *Drosophila simulans*. Ecology and Evolution 4:3330-3338.

Foerster, K., T. Coulson, B. C. Sheldon, J. M. Pemberton, T. H. Clutton-Brock, and L. E. B. Kruuk. 2007. Sexually antagonistic genetic variation for fitness in red deer. Nature 447:1107-U1109.

Fox, C. W., M. L. Bush, D. A. Roff, and W. G. Wallin. 2004. Evolutionary genetics of lifespan and mortality rates in two populations of the seed beetle, *Callosobruchus maculatus*. Heredity 92:170-181.

Gavrus-Ion, A., T. Sjovold, M. Hernandez, R. Gonzalez-Jose, M. E. E. Torne, N. Martinez-Abadias, and M. Esparza. 2017. Measuring fitness heritability: Life history traits versus morphological traits in humans. American Journal of Physical Anthropology 164:321-330.

Gay, L., E. Brown, T. Tregenza, D. Pincheira-Donoso, P. E. Eady, R. Vasudev, J. Hunt et al. 2011. The genetic architecture of sexual conflict: male harm and female resistance in *Callosobruchus maculatus*. Journal of Evolutionary Biology 24:449-456.

Griffin, R. M., H. Schielzeth, and U. Friberg. 2016. Autosomal and X-linked additive genetic variation for lifespan and aging: Comparisons within and between the sexes in *Drosophila melanogaster*. G3-Genes Genomes Genetics 6:3903-3911.

Hallsson, L. R., and M. Bjorklund. 2012. Sex-specific genetic variances in life-history and morphological traits of the seed beetle *Callosobruchus maculatus*. Ecology and Evolution 2:128-138.

Holman, L., and F. Jacomb. 2017. The effects of stress and sex on selection, genetic covariance, and the evolutionary response. Journal of Evolutionary Biology 30:1898-1909.

Innocenti, P., and E. H. Morrow. 2010. The sexually antagonistic genes of *Drosophila melanogaster*. Plos Biology 8.

Kimber, C. M., and A. K. Chippindale. 2013. Mutation, condition, and the maintenance of extended lifespan in *Drosophila*. Current Biology 23:2283-2287.

Klemme, I., and I. Hanski. 2009. Heritability of and strong single gene (Pgi) effects on life-history traits in the Glanville fritillary butterfly. Journal of Evolutionary Biology 22:1944-1953.

Kohler, H. P., J. L. Rodgers, and K. Christensen. 1999. Is fertility behavior in our genes? Findings from a Danish twin study. Population and Development Review 25:253-+.

Kosova, G., M. Abney, and C. Ober. 2010. Heritability of reproductive fitness traits in a human population. Proceedings of the National Academy of Sciences of the United States of America 107:1772-1778.

Kruuk, L. E. B., T. H. Clutton-Brock, J. Slate, J. M. Pemberton, S. Brotherstone, and F. E. Guinness. 2000. Heritability of fitness in a wild mammal population. Proceedings of the National Academy of Sciences of the United States of America 97:698-703.

Lehtovaara, A., H. Schielzeth, I. Flis, and U. Friberg. 2013. Heritability of life span is largely sex limited in *Drosophila*. American Naturalist 182:653-665.

Leips, J., and T. F. C. Mackay. 2000. Quantitative trait loci for life span in *Drosophila melanogaster*: Interactions with genetic background and larval density. Genetics 155:1773-1788.

Lewis, Z., N. Wedell, and J. Hunt. 2011. Evidence for strong intralocus sexual conflict in the Indian meal moth, *Plodia interpunctella*. Evolution 65:2085-2097.

Mallet, M. A., and A. K. Chippindale. 2011. Inbreeding reveals stronger net selection on *Drosophila melanogaster* males: implications for mutation load and the fitness of sexual females. Heredity 106:994-1002.

Martinossi-Allibert, I., G. Arnqvist, and D. Berger. 2017. Sex-specific selection under environmental stress in seed beetles. Journal of Evolutionary Biology 30:161-173.

Martinossi-Allibert, I., U. Savkovic, M. Dordevic, G. Arnqvist, B. Stojkovic, and D. Berger. 2018. The consequences of sexual selection in well-adapted and maladapted populations of bean beetles. Evolution 72:518-530.

McCleery, R. H., R. A. Pettifor, P. Armbruster, K. Meyer, B. C. Sheldon, and C. M. Perrins. 2004. Components of variance underlying fitness in a natural population of the great tit *Parus major*. American Naturalist 164:E62-E72.

McFarlane, S. E., J. C. Gorrell, D. W. Coltman, M. M. Humphries, S. Boutin, and A. G. McAdam. 2014. Very low levels of direct additive genetic variance in fitness and fitness components in a red squirrel population. Ecology and Evolution 4:1729-1738.

Merila, J., and B. C. Sheldon. 2000. Lifetime reproductive success and heritability in nature. American Naturalist 155:301-310.

Moorad, J. A., and C. A. Walling. 2017. Measuring selection for genes that promote long life in a historical human population. Nature Ecology & Evolution 1:1773-1781.

Muhlhauser, C., and W. U. Blanckenhorn. 2004. The quantitative genetics of sexual selection in the dung fly *Sepsis cynipsea*. Behaviour 141:327-341.

Pélissié, B., P. Jarne, and P. David. 2012. Sexual selection without sexual dimorphism: Bateman gradients in a simultaneous hermaphrodite. Evolution 66:66-81.

Pettay, J. E., L. E. B. Kruuk, J. Jokela, and V. Lummaa. 2005. Heritability and genetic constraints of life-history trait evolution in preindustrial humans. Proceedings of the National Academy of Sciences of the United States of America 102:2838-2843.

Poissant, J., M. B. Morrissey, A. G. Gosler, J. Slate, and B. C. Sheldon. 2016. Multivariate selection and intersexual genetic constraints in a wild bird population. Journal of Evolutionary Biology 29:2022-2035.

Punzalan, D., M. Delcourt, and H. D. Rundle. 2014. Comparing the intersex genetic correlation for fitness across novel environments in the fruit fly, *Drosophila serrata*. Heredity 112:143-148.

Qvarnstrom, A., J. E. Brommer, and L. Gustafsson. 2006. Testing the genetics underlying the co-evolution of mate choice and ornament in the wild. Nature 441:84-86.

Rapkin, J., C. R. Archer, C. E. Grant, K. Jensen, C. M. House, A. J. Wilson, and J. Hunt. 2017. Little evidence for intralocus sexual conflict over the optimal intake of nutrients for life span and reproduction in the black field cricket *Teleogryllus commodus*. Evolution 71:2159-2177.

Rodriguez-Munoz, R., A. Bretman, J. D. Hadfield, and T. Tregenza. 2008. Sexual selection in the cricket *Gryllus bimaculatus*: no good genes? Genetica 134:129-136.

Ruzicka, F., M. S. Hill, T. M. Pennell, I. Flis, F. C. Ingleby, R. Mott, K. Fowler et al. 2019. Genome-wide sexually antagonistic variants reveal long-standing constraints on sexual dimorphism in fruit flies. Plos Biology 17.

Tarka, M., M. Akesson, D. Hasselquist, and B. Hansson. 2014. Intralocus sexual conflict over wing length in a wild migratory bird. American Naturalist 183:62-73.

Teplitsky, C., J. A. Mills, J. W. Yarrall, and J. Merila. 2009. Heritability of fitness components in a wild bird population. Evolution 63:716-726.

Vermeulen, C. J., R. Bijlsma, and V. Loeschcke. 2008. A major QTL affects temperature sensitive adult lethality and inbreeding depression in life span in *Drosophila melanogaster*. Bmc Evolutionary Biology 8.

Vieira, C., E. G. Pasyukova, Z. B. Zeng, J. B. Hackett, R. F. Lyman, and T. F. C. Mackay. 2000. Genotype-environment interaction for quantitative trait loci affecting life span in *Drosophila melanogaster*. Genetics 154:213-227.

Walling, C. A., M. B. Morrissey, K. Foerster, T. H. Clutton-Brock, J. M. Pemberton, and L. E. B. Kruuk. 2014. A multivariate analysis of genetic constraints to life history evolution in a wild population of red deer. Genetics 198:1735-+.

Wayne, M. L., J. B. Hackett, C. L. Dilda, S. V. Nuzhdin, E. G. Pasyukova, and T. F. C. MacKay. 2001. Quantitative trait locus mapping of fitness-related traits in *Drosophila melanogaster*. Genetical Research 77:107-116.

Wheelwright, N. T., L. F. Keller, and E. Postma. 2014. The effect of trait type and strength of selection on heritability and evolvability in an island bird population. Evolution 68:3325-3336.

Wolak, M. E., P. Arcese, L. F. Keller, P. Nietlisbach, and J. M. Reid. 2018. Sex-specific additive genetic variances and correlations for fitness in a song sparrow (*Melospiza melodia*) population subject to natural immigration and inbreeding. Evolution 72:2057-2075.

Zajitschek, F., J. Hunt, S. R. K. Zajitschek, M. D. Jennions, and R. Brooks. 2007. No intra-locus sexual conflict over reproductive fitness or ageing in field crickets. Plos One 2.

Zietsch, B. P., R. Kuja-Halkola, H. Walum, and K. J. H. Verweij. 2014. Perfect genetic correlation between number of offspring and grandoffspring in an industrialized human population. Proceedings of the National Academy of Sciences of the United States of America 111:1032-1036.
