# Supplementary material for: Stronger net selection on males across animals
Source: eLife. 2021 Nov 17;10:e68316. doi: 10.7554/eLife.68316 (PMC8598160; doi:10.7554/eLife.68316)
Supplement: Supplementary file 9. [file elife-68316-supp9.docx]

**Supplementary File 9. Methodological characteristics of the 55 primary studies.** Table shows for each study the species’ Latin name, the number of estimates (*N*), the fitness component (**RS**: reproductive success; **LS**: lifespan), the study type (**lab**oratory studies; **field** studies), the breeding design, the estimate of genetic variance *V*_G_ (**additive** genetic variance; **total** genetic variance including additive variance, dominance variance, and epistatic variance), the type of reproductive success metric RS (**RS**: reproductive success measured for a limited time period; **LRS**: lifetime reproductive success), the specification of RS (**ad**: number of adult offspring; **grand**: number of grand-offspring; **juv**: number of juvenile offspring; **sex-spec**: sex-specific measures of reproductive success), and paternity assessment (**Phe**: phenotyping offspring based on genetic marker; **Gen**: genotyping offspring; **Beh**: behavioral observations; **Ste**: sterile male technique; **Soc**: social parentage; **NoC**: no competition making paternity assessment unnecessary for estimating reproductive success). See methods section for further details.

| Study | Species | *N* | Fitness compo-nent | Study type | Breeding design | Estimate of *V*_G_ | Type of RS | Specification  of RS | Paternity assess-ment |
| --- | --- | --- | --- | --- | --- | --- | --- | --- | --- |
| Abbott and Nordén (in prep.) | *Macrostomum lignano* | 1 | RS | Lab | Half-sib | additive | RS | juv | Phe |
| Archer et al. 2012 | *Gryllodes sigillatus* | 1 | LS | Lab | Lines | total | - | - | - |
| Berger et al. 2014 | *Callosobruchus maculatus* | 4 | RS | Lab | Lines | total | LRS | ad | Ste |
| Bolund et al. 2013 | *Homo sapiens* | 2 | RS, LS | Field | Pedigree | additive | LRS | grand | Soc |
| Brommer et al. 2007 | *Ficedula albicollis* | 1 | RS | Field | Pedigree | additive | LRS | ad | Soc |
| Calsbeek et al. 2015 | *Anolis sagrei* | 1 | RS | Field | Pedigree | additive | RS | ad | Gen |
| Collet et al. 2016 | *Drosophila melanogaster* | 1 | RS | Lab | Lines | total | RS | ad | Phe |
| Coltman et al. 2005 | *Ovis canadensis* | 2 | RS, LS | Field | Pedigree | additive | LRS | ad | Gen |
| Delcourt et al. 2009 | *Drosophila serrata* | 2 | RS | Lab | Half-sib | additive | RS | ad | Phe |
| Duffy et al. 2014 | *Drosophila simulans* | 1 | RS | Lab | Lines | total | RS | ad | Phe |
| Duffy et al. 2019 | *Drosophila simulans* | 2 | RS | Lab | Lines | total | RS | ad | Phe |
| Foerster et al. 2007 | *Cervus elaphus* | 1 | RS | Field | Pedigree | additive | RS | ad | Gen, Beh |
| Fox et al. 2004 | *Callosobruchus maculatus* | 4 | LS | Lab | Half-sib | additive | - | - | - |
| Gavrus-Ion et al. 2017 | *Homo sapiens* | 2 | RS, LS | Field | Pedigree | additive | RS | ad | Soc |
| Gay et al. 2010 | *Callosobruchus maculatus* | 2 | RS, LS | Lab | Half-sib | additive | LRS | ad | Noc |
| Griffin et al. 2016 | *Drosophila melanogaster* | 1 | LS | Lab | Lines | total | - | - | - |
| Hallsson and Björklund 2012 | *Callosobruchus maculatus* | 1 | LS | Lab | Half-sib | additive | - | - | - |
| Holman and Jacomb 2017 | *Tribolium castaneum* | 2 | RS | Lab | Half-sib | additive | RS | sex-spec | Noc |
| Innocenti and Morrow 2010 | *Drosophila melanogaster* | 1 | RS | Lab | Lines | total | LRS | ad | Phe |
| Janicke et al. (in prep.) | *Physa acuta* | 2 | RS | Lab | Full-sib | total | RS | juv | Phe |
| Kimber and Chippindale 2013 | *Drosophila melanogaster* | 2 | RS, LS | Lab | Lines | total | RS | ad | Phe |
| Klemme and Hanski 2009 | *Melitaea cinxia* | 1 | LS | Lab | Full-sib | total | - | - | - |
| Kohler et al. 1999 | *Homo sapiens* | 1 | RS | Field | Twin study | total | LRS | juv | Soc |
| Kosova et al. 2010 | *Homo sapiens* | 1 | RS | Field | Pedigree | additive | LRS | juv | Soc |
| Kruuk et al. 2000 | *Cervus elaphus* | 2 | RS, LS | Field | Pedigree | additive | LRS | juv | Gen, Beh |
| Lehtovaara et al. 2013 | *Drosophila melanogaster* | 2 | LS | Lab | Lines | total | - | - | - |
| Leips and Mackay 2000 | *Drosophila melanogaster* | 4 | LS | Lab | Lines | total | - | - | - |
| Lewis et al. 2011 | *Plodia interpunctella* | 1 | LS | Lab | Half-sib | additive | - | - | - |
| Mallet and Chippendale 2011 | *Drosophila melanogaster* | 1 | RS | Lab | Lines | total | RS | ad | Phe |
| Martinossi-Allibert et al. 2017 | *Callosobruchus maculatus* | 5 | RS | Lab | Lines | total | LRS | ad | Ste |
| Martinossi-Allibert et al. 2018 | *Acanthoscelides obtectus* | 4 | RS | Lab | Lines | total | LRS | ad | Ste |
| McCleery et al. 2004 | *Parus major* | 2 | RS, LS | Field | Pedigree | additive | LRS | ad | Soc |
| McFarlane et al. 2013 | *Tamiasciurus hudsonicus* | 1 | RS | Field | Pedigree | additive | LRS | juv | Gen |
| Merila and Sheldon 2000 | *Ficedula albicollis* | 2 | RS, LS | Field | Pedigree | additive | LRS | ad | Soc |
| Moiron et al. (in prep.) | *Sterna hirundo* | 2 | RS, LS | Field | Pedigree | additive | LRS | juv | Soc |
| Moorad and Walling 2017 | *Homo sapiens* | 2 | RS, LS | Field | Pedigree | additive | LRS | juv | Soc |
| Mühlhäuser and Blanckenhorn 2003 | *Sepsis cynipsea* | 1 | LS | Lab | Half-sib | additive | - | - | - |
| Pelissie et al. 2012 | *Physa acuta* | 1 | RS | Lab | Full-sib | total | RS | juv | Phe |
| Pettay et al. 2005 | *Homo sapiens* | 2 | RS, LS | Field | Pedigree | additive | RS | juv | Soc |
| Poissant et al. 2016 | *Parus major* | 2 | RS, LS | Field | Pedigree | additive | LRS | ad | Soc |
| Punzalan et al. 2014 | *Drosophila serrata* | 4 | RS | Lab | Lines | total | RS | ad | Phe |
| Qvarnström et al. 2006 | *Ficedula albicollis* | 1 | RS | Field | Pedigree | additive | RS | ad | Soc |
| Rapkin et al. 2017 | *Teleogryllus commodus* | 4 | RS | Lab | Half-sib | additive | RS | juv | Noc |
| Rodriguez-Munoz et al. 2008 | *Gryllus bimaculatus* | 1 | LS | Lab | Half-sib | additive | - | - | - |
| Ruzicka et al. 2019 | *Drosophila melanogaster* | 1 | RS | Lab | Lines | total | RS | sex-spec | Phe |
| Tarka et al. 2014 | *Acrocephalus arundinaceus* | 1 | RS | Field | Pedigree | additive | LRS | juv | Gen, Beh |
| Teplitsky et al. 2009 | *Larus novaehollandiae* | 2 | RS, LS | Field | Pedigree | additive | RS | ad | Soc |
| Vermeulen et al. 2008 | *Drosophila melanogaster* | 1 | LS | Lab | Half-sib | additive | - | - | - |
| Vieira et al. 2000 | *Drosophila melanogaster* | 5 | LS | Lab | Lines | total | - | - | - |
| Walling et al. 2014 | *Cervus elaphus* | 2 | RS, LS | Field | Pedigree | additive | RS | juv | Gen, Beh |
| Wayne et al. 2001 | *Drosophila melanogaster* | 1 | RS | Lab | Lines | total | RS | ad | Phe |
| Wheelwright et al. 2014 | *Passerculus sandwichensis* | 2 | RS, LS | Field | Pedigree | additive | LRS | juv | Gen |
| Wolak et al. 2018 | *Melospiza melodia* | 1 | RS | Field | Pedigree | additive | RS | ad | Gen |
| Zajitschek et al. 2007 | *Teleogryllus commodus* | 1 | LS | Lab | Half-sib | additive | - | - | - |
| Zietsch et al. 2014 | *Homo sapiens* | 1 | RS | Field | Twin study | total | LRS | juv | Soc |
