# Supplementary material for: Stronger net selection on males across animals
Source: eLife. 2021 Nov 17;10:e68316. doi: 10.7554/eLife.68316 (PMC8598160; doi:10.7554/eLife.68316)
Supplement: Supplementary file 10. [file elife-68316-supp10.docx]

**Supplementary File 10.** Mating system classification (monogamy versus polygamy) of the 26 sampled species in alphabetical order.

| Species | Class | Mating System | Reference |
| --- | --- | --- | --- |
| *Acanthoscelides obtectus* | Insecta | Polygamy | (Seslija *et al.* 2009) |
| *Acrocephalus arundinaceus* | Aves | Polygamy | (Hasselquist *et al.* 1995) |
| *Anolis sagrei* | Reptilia | Polygamy | (Kamath & Losos 2018) |
| *Callosobruchus maculatus* | Insecta | Polygamy | (Fritzsche & Arnqvist 2013) |
| *Cervus elaphus* | Mammalia | Polygamy | (Bonenfant *et al.* 2004; Frantz *et al.* 2008) |
| *Drosophila melanogaster* | Insecta | Polygamy | (Morimoto *et al.* 2016) |
| *Drosophila serrata* | Insecta | Polygamy | (Frentiu & Chenoweth 2008) |
| *Drosophila simulans* | Insecta | Polygamy | (Taylor *et al.* 2008) |
| *Ficedula albicollis* | Aves | Monogamy | (Qvarnstrom *et al.* 2003) |
| *Gryllodes sigillatus* | Insecta | Polygamy | (Ivy & Sakaluk 2005) |
| *Gryllus bimaculatus* | Insecta | Polygamy | (Bretman & Tregenza 2005) |
| *Homo sapiens* | Mammalia | Monogamy | (Dixson 2009) |
| *Larus novaehollandiae* | Aves | Monogamy | (Mills 1973) |
| *Macrostomum lignano* | Rhabditophora | Polygamy | (Janicke & Schärer 2009) |
| *Melitaea cinxia* | Insecta | Polygamy | (Boggs & Nieminen 2004) |
| *Melospiza melodia* | Aves | Monogamy | (Arcese 1989) |
| *Ovis canadensis* | Mammalia | Polygamy | (Coltman *et al.* 2002) |
| *Parus major* | Aves | Monogamy | (Dhondt 1987) |
| *Passerculus sandwichensis* | Aves | Polygamy | (Wheelwright *et al.* 1992) |
| *Physa acuta* | Gastropoda | Polygamy | (Pélissié *et al.* 2012) |
| *Plodia interpunctella* | Insecta | Polygamy | (Gage 1995) |
| *Sepsis cynipsea* | Insecta | Polygamy | (Teuschl & Blanckenhorn 2007) |
| *Sterna hirundo* | Aves | Monogamy | (Griggio *et al.* 2004) |
| *Tamiasciurus hudsonicus* | Mammalia | Polygamy | (Lane *et al.* 2008) |
| *Teleogryllus commodus* | Insecta | Polygamy | (Evans 1988; Jennions *et al.* 2004) |
| *Tribolium castaneum* | Insecta | Polygamy | (Fedina & Lewis 2008) |

**References – Mating system classification**

Arcese, P. (1989). Intrasexual competition, mating system and natal dispersal in song sparrows. *Anim. Behav.*, 38, 958-979.

Boggs, C. & Nieminen, M.J. (2004). Cherckerspot reproductive biology. In: *On the wings of checkerspots: A model system for population biology*. Oxford University Press, pp. 92-111.

Bonenfant, C., Gaillard, J.M., Klein, F. & Maillard, D. (2004). Variation in harem size of red deer (*Cervus elaphus* L.): the effects of adult sex ratio and age-structure. *J. Zool.*, 264, 77-85.

Bretman, A. & Tregenza, T. (2005). Measuring polyandry in wild populations: a case study using promiscuous crickets. *Mol. Ecol.*, 14, 2169-2179.

Coltman, D.W., Festa-Bianchet, M., Jorgenson, J.T. & Strobeck, C. (2002). Age-dependent sexual selection in bighorn rams. *Proc. R. Soc. B-Biol. Sci.*, 269, 165-172.

Dhondt, A.A. (1987). Polygynous blue tits and monogamous great tits: does the polygyny-threshold model hold? *Am. Nat.*, 129, 213-220.

Dixson, A.F. (2009). *Sexual selection and the origins of human mating systems*. Oxford University Press.

Evans, A. (1988). Mating systems and reproductive strategies in three australian gryllid crickets: *Bobilla victoriae* Otte, *Balamara gidya* Otte and *Teleogryllus commodus* (Walker) (Orthoptera: Gryllidae: Nemobiinae; Trigonidiinae; Gryllinae). *Ethology*.

Fedina, T.Y. & Lewis, S.M. (2008). An integrative view of sexual selection in Tribolium flour beetles. *Biol. Rev.*, 83, 151-171.

Frantz, A.C., Hamann, J.L. & Klein, F. (2008). Fine-scale genetic structure of red deer (*Cervus elaphus*) in a French temperate forest. *European Journal of Wildlife Research*, 54, 44-52.

Frentiu, F.D. & Chenoweth, S.F. (2008). Polyandry and paternity skew in natural and experimental populations of *Drosophila serrata*. *Mol. Ecol.*, 17, 1589-1596.

Fritzsche, K. & Arnqvist, G. (2013). Homage to Bateman: sex roles predict sex differences in sexual selection. *Evolution; international journal of organic evolution*, 67, 1926-1936.

Gage, M.J. (1995). Continuous variation in reproductive strategy as an adaptive response to population density in the moth *Plodia interpunctella*. *Proceedings of the Royal Society of London. Series B: Biological Sciences*, 261, 25-30.

Griggio, M., Matessi, G. & Marin, G. (2004). No evidence of extra-pair paternity in a colonial seabird, the common tern (*Sterna hirundo*). *Ital. J. Zool.*, 71, 219-222.

Hasselquist, D., Bensch, S. & Vonschantz, T. (1995). Low frequency of extrapair paternity in the polygynous great reed warbler, *Acrocephalus arundinaceus*. *Behav. Ecol.*, 6, 27-38.

Ivy, T.M. & Sakaluk, S.K. (2005). Polyandry promotes enhanced offspring survival in decorated crickets. *Evolution*, 59, 152-159.

Janicke, T. & Schärer, L. (2009). Determinants of mating and sperm-transfer success in a simultaneous hermaphrodite. *Journal of Evolutionary Biology*, 22, 405-415.

Jennions, M.D., Hunt, J., Graham, R. & Brooks, R. (2004). No evidence for inbreeding avoidance through postcopulatory mechanisms in the black field cricket, *Teleogryllus commodus*. *Evolution*, 58, 2472-2477.

Kamath, A. & Losos, J.B. (2018). Estimating encounter rates as the first step of sexual selection in the lizard *Anolis sagrei*. *Proc. R. Soc. B-Biol. Sci.*, 285, 9.

Lane, J.E., Boutin, S., Gunn, M.R., Slate, J. & Coltman, D.W. (2008). Female multiple mating and paternity in free-ranging North American red squirrels. *Anim. Behav.*, 75, 1927-1937.

Mills, J.A. (1973). The influence of age and pair-bond on the breeding biology of the red-billed gull *Larus novaehollandiae scopulinus*. *J. Anim. Ecol.*, 42, 147-162.

Morimoto, J., Pizzari, T. & Wigby, S. (2016). Developmental environment effects on sexual selection in male and female *Drosophila melanogaster*. *PLoS One*, 11, 27.

Pélissié, B., Jarne, P. & David, P. (2012). Sexual selection without sexual dimorphism: Bateman gradients in a simultaneous hermaphrodite. *Evolution*, 66, 66-81.

Qvarnstrom, A., Sheldon, B.C., Part, T. & Gustafsson, L. (2003). Male ornamentation, timing of breeding, and cost of polygyny in the collared flycatcher. *Behav. Ecol.*, 14, 68-73.

Seslija, D., Lazarevic, J., Jankovic, B. & Tucic, N. (2009). Mating behavior in the seed beetle *Acanthoscelides obtectus* selected for early and late reproduction. *Behav. Ecol.*, 20, 547-552.

Taylor, M.L., Wigmore, C., Hodgson, D.J., Wedell, N. & Hosken, D.J. (2008). Multiple mating increases female fitness in *Drosophila simulans*. *Anim. Behav.*, 76, 963-970.

Teuschl, Y. & Blanckenhorn, W.U. (2007). The reluctant fly: what makes *Sepsis cynipsea* females willing to copulate? *Anim. Behav.*, 73, 85-97.

Wheelwright, N.T., Schultz, C.B. & Hodum, P.J. (1992). Polygyny and male parental care in Savannah sparrows: effects on female fitness. *Behav. Ecol. Sociobiol.*, 31, 279-289.
