# Supplementary material for: Stronger net selection on males across animals
Source: eLife. 2021 Nov 17;10:e68316. doi: 10.7554/eLife.68316 (PMC8598160; doi:10.7554/eLife.68316)
Supplement: Supplementary file 11. [file elife-68316-supp11.docx]

**Supplementary File 11. Phylogenetically independent meta-analysis of lnCVR defined as the log-transformed ratio of male to female coefficient of phenotypic variation.** Models shown are testing for (a) the intercept across mating systems (i.e., global effect size with positive values indicating a male bias), (b) with mating system as moderator variable and separately for (c) monogamous and (d) polygamous species. Results are shown for reproductive success (RS) and lifespan (LS). Estimates are shown as posterior means with 95% Highest Posterior Density (HPD) intervals. *P*_MCMC_ is the probability of the posteriors including zero.

| Response | Predictor | Estimate | | | *P*_MCMC_ |
| --- | --- | --- | --- | --- | --- |
| (a) Across mating systems | |  |  |  |  |
| RS | Intercept | 0.232 | (-0.249, | 0.705) | 0.257 |
| LS | Intercept | 0.030 | (-0.094, | 0.151) | 0.576 |
| (b) Mating system | |  |  |  |  |
| RS | Intercept | -0.032 | (-0.360, | 0.266) | 0.835 |
|  | Mating system | 0.363 | (0.124, | 0.624) | 0.011 |
| LS | Intercept | 0.082 | (-0.107, | 0.275) | 0.357 |
|  | Mating system | -0.077 | (-0.270, | 0.129) | 0.436 |
| (c) Monogamy |  |  |  |  |  |
| RS | Intercept | 0.056 | (-1.336, | 1.470) | 0.791 |
| LS | Intercept | 0.064 | (-0.325, | 0.454) | 0.508 |
| (d) Polygamy |  |  |  |  |  |
| RS | Intercept | 0.326 | (0.007, | 0.612) | 0.043 |
| LS | Intercept | 0.006 | (-0.166, | 0.192) | 0.927 |
